# Healthcare professionals’ representation toward optimal palliative care provision for COPD patients: a cross-sectional survey

**DOI:** 10.1177/17534666251341748

**Published:** 2025-05-28

**Authors:** Filipa Alexandra Baptista Peixoto Befecadu, Paola Gasche, Dan Adler, Ivan Guerreiro, Sophie Pautex, Lisa Hentsch

**Affiliations:** Research and Implementation Fellow, Research and Implementation Care Lab, Care Directorate, Geneva University Hospitals, Geneva Bd de la Tour 8, 1205 Geneva, Switzerland; Division of Pneumology, Department of Medicine, Geneva University Hospitals, Geneva, Switzerland; Division of Pulmonary Diseases, Hôpital de la Tour, Geneva, Switzerland; Division of Pneumology, Department of Medicine, Geneva University Hospitals, Geneva, Switzerland; Division of Palliative Medicine, Department of Readaptation and Geriatrics, Geneva University Hospitals and Geneva University, Geneva, Switzerland; Division of Palliative Medicine, Department of Readaptation and Geriatrics, Geneva University Hospitals, Geneva, Switzerland

**Keywords:** chronic obstructive pulmonary disease, healthcare professionals, palliative care, quality of life

## Abstract

**Background:**

Chronic obstructive pulmonary disease (COPD) is a condition causing chronic physical symptoms, psychological burdens, as well as social consequences. This contributes to a major decrease in quality of life (QoL). Palliative care (PC) is a person-centered approach intended to relieve physical, psychological, social, and spiritual suffering. Despite international practice guidelines, patients with COPD have limited access to PC, mostly during end-of-life (EoL) care. It is therefore important to explore healthcare professionals’ (HPs) point of view about PC to improve access for COPD patients to PC.

**Objectives:**

This study aimed to describe the perceptions of HPs working with COPD patients in Switzerland in different settings on PC provision, implementation, access, and organization. Additionally, we aimed to identify gaps, barriers, training needs, and solutions for HPs related to PC needs.

**Design:**

This is a cross-sectional survey study with quantitative and open-ended questions.

**Methods:**

We used an electronic survey sent to HPs working with patients suffering from COPD in the inpatient, outpatient, and home-based settings.

**Results:**

A total of 56 out of 98 participants (57%) answered the questionnaire of which 41.1% were nurses. 47.2% of participants were uncertain about the good timing of addressing COPD patients to PC and did it after several acute exacerbations, during EoL, or at the request of the patient. 45.5% did not know the availability of a local specialized PC. Lack of skills/training was identified as one of the hindering factors to discuss EoL (42.9% *N* = 56).

**Conclusion:**

Despite recognizing the importance of PC, several barriers were identified, including a lack of knowledge about when to initiate a PC and limited utilization of tools for identifying PC needs. Multidisciplinary teamwork and the identification of a nurse coordinator could improve earlier referrals to PC and improve QoL for COPD patients.

## Introduction

In Switzerland, more than 400,000 people suffer from chronic obstructive pulmonary disease (COPD).^1,2^ COPD is a major cause of morbidity and mortality in high-income countries and is the fourth cause of death worldwide.^3–7^ In addition to physical symptoms like persistent dyspnea, fatigue, and psychological burdens such as anxiety and depression or social isolation, COPD patients experience feelings of meaninglessness and guilt.^8–10^ This contributes to a major decrease in quality of life (QoL) over the course of the illness trajectory.^11,12^

Palliative care (PC) is a person-centered approach intended to relieve physical, psychological, social, and spiritual suffering to improve QoL.^13^ Specialist PC is PC delivered by healthcare professionals (HPs) with a degree in PC, as opposed to general PC, which is delivered by nonspecialists HPs.^14^ The introduction of PC in the early stages of life-limiting diseases helps to prevent suffering and promotes QoL.^15,16^ Despite proven benefits and being recommended by major national and international societies, patients with COPD have only limited access to PC in the outpatient, inpatient, and home-based settings and is generally only introduced late in the disease trajectory, mainly as end-of-life (EoL) care.^4,17–20^ The uncertainty of the disease trajectory makes it difficult for HPs to identify the “right time” to refer COPD patients to PC and the exact timing remains subjective despite the proposal of referral criteria.^19,21,22^ Misconceptions about PC as exclusive EoL care among patients and HPs have been identified as barriers to the early implementation of PC.^17,18^

The Global Strategy for the Diagnosis, Management, and Prevention of Chronic Obstructive Lung Disease (GOLD) recommends that patients should be informed that they may become seriously ill, and they and/or their relatives should decide whether intensive care treatments suit their goals of care and personal values.^23^ Furthermore, it encourages HPs to start conversations about these possible scenarios while patients are still able to make informed decisions.^23^ Discussions about advance care planning (ACP) or EoL decisions are reported to be difficult topics to address with patients living with COPD.^4,24^ Previous research shows that the most frequent reasons to avoid these discussions were (a) the clinicians’ feeling that their patients were not ready for these discussions, (b) the fear of destroying the patient’s hope, (c) insufficient training, (d) lack of clarity about the right timing, and (e) lack of confidence.^24–26^ Although most physicians do discuss the progression of the disease with their patients, life expectancy, suffering, and other questions related to dying are less frequently addressed.^24^ Pneumologists, respiratory specialized nurses, respiratory therapists, and other HPs who are already caring for patients living with COPD are in the best position to recognize and identify PC needs. A multidisciplinary approach is needed to address COPD’s high symptom burden, frequent exacerbations, and unpredictable disease trajectories.^20^ Furthermore, multidisciplinary teamwork enhances communication, ensures continuity of care, and enables early identification and management of PC needs, which are often under-recognized in COPD. This integrative approach not only improves QoL but also supports caregivers, ultimately leading to more effective and compassionate care delivery.^27^

Switzerland’s healthcare system is a decentralized, high-quality model that emphasizes individual responsibility and universal access. All residents are required to purchase basic health insurance, ensuring a high level of patient choice and fostering competition among providers.^28^ Healthcare services are managed at both federal and cantonal levels, with cantons financing and organizing local healthcare delivery. This structure emphasizes outpatient care, with general practitioners playing a central role, supported by both public and private hospitals offering acute, rehabilitation, and psychiatric care.^29^

The aim of this study was to describe the perceptions of HPs in the Geneva area (Switzerland) working with patients living with COPD on PC provision, implementation, access, and organization. Our secondary objective was to identify gaps, barriers, and potential information and training needs of HPs related to the PC needs of patients with advanced COPD.

## Materials and methods

### Study design

This cross-sectional survey used an online questionnaire conducted in relation to PC for patients with COPD. The online survey was disseminated using Research Electronic Data Capture (REDCap^®^) version 10.0.33 developed and hosted by Vanderbilt University, Nashville, TN, USA. We followed the CHERRIES reporting guidelines to ensure comprehensive reporting and adherence to rigorous standards for studies involving electronic surveys.^30^

### Setting

In Switzerland, respiratory diseases, including COPD, are typically managed by a combination of pneumologists, general practitioners, specialized nurses, and physiotherapists. In Geneva, a Swiss French-speaking canton with a population of approximately 506,343 people and the second most populated city in Switzerland, 26 registered pneumologists worked in the outpatient/community setting and 12 in a hospital setting in 2023.^31–33^ Specialized respiratory nurses worked in hospitals, rehabilitation, and the outpatient setting, particularly at institutions like the Geneva University Hospital. An estimated 15–25 nurses are dedicated to respiratory care, including COPD management, within larger institutions. The number of HPs working for the *Ligue Pulmonaire Genevoise* (a nonprofit health organization with the aim of promoting lung health and improving the QoL for people affected by lung diseases in Switzerland) was 12.

This study included HPs from three different settings: the Geneva University Hospitals, the *Ligue Pulmonaire Genevoise*, and pneumologists working in the community.

### Participants

We used purposive sampling to recruit participants. A total of 98 HPs were invited to participate. The only eligibility criterion was that participants had to be working closely with patients living with COPD. All board-certified pneumologists, respiratory nurses (including respiratory specialized nurses and registered nurses), nurse assistants, and care assistants working in the Pulmonary division of the Geneva University Hospitals were invited, as well as nurses and respiratory therapists working for the Geneva division of the *Ligue Pulmonaire*. All pneumologists, members of the *Association des Médecins du Canton de Genève* (AMGe), working in private practice in the Geneva area, were invited.

### Outcome

We developed a self-administered survey for this study (see Supplemental Material—Appendix 1). Questions were drawn after a review of the existing literature and tested by a sample of three participants.^24,34–38^ The survey comprises a sociodemographic section followed by four other sections. The sociodemographic section included questions related to: age, gender, profession, seniority, and experience in PC. The four other sections were divided into: (1) identification of PC needs, (2) perceptions of PC for patients with COPD, (3) information and content delivered to patients with COPD, and (4) organization of care for COPD patients. In each section, questions were either multiple-choice, ranking (e.g., never, rarely, often, most of the time, and almost always), or open-ended with adaptive questioning. Questions were also adapted according to the profession of each participant. A completeness check was performed at the end of each page and participants could go back to previous pages to review or change their answers. The total number of questions was 31 distributed over 5 pages, each representing one section. The time for completion of the questionnaire took approximately 10–15 min.

### Study procedure

An email invitation was sent to all participants with information about participation and a direct link to the online questionnaire which then collected data automatically in a protected REDCap^®^ without the possibility to link the answered surveys to a specific email. Reminders were sent after 3, 5, and 8 weeks to remind nonresponders to participate (automatically generated by REDCap^®^). Eligible participants were free to decline participation by not responding to the email. No incentives were offered for participation in the study. Participants responded to the online questionnaire between August and October 2021.

### Data management and analysis

Upon completion of the survey, the responses were automatically captured and securely stored in a REDCap^®^ database. To ensure confidentiality and privacy, personal identifiers, such as names and email addresses, were not collected or stored.

The collected survey data underwent a thorough cleaning process to ensure data quality and integrity. This involved checking for missing values and inconsistencies. Any incomplete or invalid responses were excluded from the analysis. The data were then prepared for analysis by assigning numerical codes or categories to the multiple-choice responses and organizing the open-ended responses for content analysis.^39^

Descriptive statistics were performed using the STATA 14.2 (StataCorp LLC, College Station, TX, USA) software. Frequencies, percentages, means, and standard deviations were calculated to summarize and describe data. Open-ended questions were analyzed using qualitative content analysis to identify recurring themes and patterns related to each section. Most of the open-ended questions had short responses. The responses were first read and re-read to become familiar with the content, then systematically coded and categorized into themes. A content analysis was conducted to identify and summarize the key patterns and insights that emerged from the data.^39^ This process involved two members of the research team with experience in qualitative research (FB, LH).

## Results

### Sociodemographic data

Of the 98 HPs solicited, a total of 56 participants (57%) answered the online survey; the mean age was 44.9 years (9.96 SD). The sample’s demographic characteristics are presented in Table 1.

**Table 1.** Demographic characteristics.

Variables	*N*	Percent
Sex		
Male	27	48.2
Female	28	50.0
Other	1	1.8
Profession		
Pneumologist	25	44.6
Respiratory therapist	2	3.6
Nurse	23	41.1
Nurse assistant	2	3.6
Care assistant	4	7.1
Work context		
Inpatient	31	55.4
Outpatient	7	12.5
Private practice	9	16.1
Lung organization	8	14.3
Other	1	1.8
Years of experience		
<1 year	1	1.8
1–3 years	7	12.5
3–5 years	10	17.9
>5 years	38	67.9
Training in palliative care		
Yes	7	12.5
No	49	87.5
Worked in a palliative care unit in the past		
Yes	7	12.5
No	49	87.5

### Identification of palliative care needs

Thirty-eight participants (70.4%, *N* = 54) agreed that patients living with COPD require PC, while 15 (27.8%, *N* = 54) participants disagreed with this idea. In the open-ended question concerning the main reasons why participants thought that patients with COPD require PC, the main answers were centered around the idea that COPD is a silent, progressive, disabling disease that causes physical, psychological, and social suffering. Participants mainly mentioned that: (a) COPD has a significant impact on the functional level and on the patient’s experience, (b) the prognosis of COPD is unfavorable and is associated with a decrease in life expectancy and (c) the disease has troublesome symptoms such as dyspnea and anxiety that lead to recurrent hospitalizations.

Regarding participants’ perceptions of the benefits of PC for COPD patients, most participants (94.6%, *N* = 56) agreed that PC helps in alleviating physical, psychological, and spiritual symptoms in COPD patients (see Table 2).

**Table 2.** Participants’ perceptions of the benefits of palliative care.

In your opinion, palliative care can help:	*N* = 56	Percent
Alleviate physical, psychological, and spiritual symptoms	53	94.6
Improve quality of life and have a positive influence on the trajectory of the disease	42	75
Initiate discussions on the advance care plan	31	55.4
Provide support to caregivers (respite/education)	31	55.4
Managing the end-of-life phase	26	46.4
Discuss prognosis	9	16.1
Improve patients’ understanding of the disease	9	16.1

The answer to the question “Do you know when to initiate a palliative care approach/management in COPD” was “No” for 25 (47.2%, *N* = 53) participants; “Yes” for 19 (35.8%, *N* = 53) and “Maybe” for 9 (17%, *N* = 53). Among those who answered “No” (*n* = 25), the main reason was a lack of knowledge about when, where, and how to refer patients to specialist PC. Participants who answered “Yes” (*n* = 19) or “Maybe” (*n* = 9) initiated PC mostly when patients asked for it or when the symptoms were not relieved enough.

Forty-three participants (89.6%, *N* = 48) do not use guidelines/scales/tools to identify the PC needs of COPD patients. They reported that they did not know the existence of such tools, or that they did not master them. In some cases, they said they did not have time to use it. Five participants (10.4%, *N* = 48) use the Edmonton Symptom Assessment System (ESAS),^40^ the COPD Assessment Test (CAT),^41^ the Body mass index, airflow Obstruction, Dyspnea, and Exercise capacity (BODE),^42^ the Supportive & Palliative Care Indicators Tool (SPICT™)^43^ and/or the Pallia 10 CH.^44^

Our participants, especially physicians, respiratory therapists, and nurses, were asked to rate the criteria used in their practice to identify the need for PC in COPD patients from “Never” to “Always” (see Appendix 2). The most frequently mentioned criteria (stated as always) were the desire expressed by the patient (*n* = 24, 53.3%), repeated hospital admission for COPD exacerbation (n = 11, 23.9%), and clinical experience (*n* = 21, 45.7%). Another criterion used in clinical practice to identify the need for PC in COPD patients was the social context of the patient.

The most frequently observed symptoms in COPD rated “Almost always” (see Table 3) were dyspnea (*n* = 28, 58.3%), fatigue (n = 17, 35.4%), and anxiety (*n* = 16, 33.3%). Other symptoms frequently mentioned in the open-ended question were sleep disorders, irritation, impatience, cough, lack of appetite, cachexia, undernutrition, sense of frustration, isolation, and impact on activities of daily living. Other signs were muscular weakness, difficulty in mobilization, bronchorrhea, cognitive disorders, cyanosis, and undernutrition.

**Table 3.** Frequency of common symptoms in patients with COPD.

How often do you observe the following symptoms in patients with COPD?	Never	Rarely (<25%)	Often (25%–50%)	Most of the time (50%–75%)	Almost always (>75%)
**Dyspnea *N* = 48**	0 (0%)	0 (0%)	1 (2.1%)	19 (39.6%)	28 (58.3%)
**Pain *N* = 48**	1 (2.1%)	22 (45.8%)	19 (39.6%)	5 (10.4%)	1 (2.1%)
**Anxiety *N* = 48**	0 (0%)	0 (0%)	12 (25%)	20 (41.7%)	16 (33.3%)
**Depression *N* = 48**	0 (0%)	1 (2.1%)	29 (60.4%)	15 (31.3%)	3 (6.3%)
**Fatigue *N* = 48**	0 (0%)	0 (0%)	14 (29.2%)	17 (35.4%)	17 (35.4%)
**Nausea *N* = 48**	11 (22.9%)	30 (62.5%)	7 (14.6%)	0 (0%)	0 (0%)
**Drowsiness *N* = 48**	3 (6.3%)	24 (50%)	18 (37.5%)	2 (4.2%)	1 (2.1%)
**Other *N* = 44**	30 (68.2%)	2 (4.6%)	7 (15.9%)	5 (11.4%)	0 (0%)

COPD, Chronic obstructive pulmonary disease.

Participants were asked to rate how often they refer their COPD patients to specialized PC services at different specific moments of the trajectory of the disease (Table 4). The other times mentioned in the open-ended question were (a) if the patient and/or informal caregiver requested PC, (b) if patient had significant discomfort and distress, (c) exacerbated dyspnea, (d) repeated exacerbation of other comorbidities, (e) multiple disabling symptoms, the (f) impossibility to be discharged from hospital, (g) major decrease in his/her general condition between admission and discharge from hospital, (h) admission of the patient in a poor general condition (hygiene, etc.), suggesting that he/she can no longer provide for his/her basic needs alone, or (i) when the situation is complex if a patient is very limited in his/her activities due to dyspnea.

**Table 4.** Referral practices for COPD patients to specialized palliative care services.

When do you refer COPD patients to specialized palliative care (outpatient/hospital/home)? *N* = 44					
	Never	Rarely (<25%)	Often (25%–50%)	Most of the time (50%–75%)	Almost always (>75%)
**At the time of diagnosis**	29 (65.9%)	14 (31.8%)	1 (2.3%)	0 (0%)	0 (0%)
**After the first acute exacerbation**	25 (56.8%)	17 (38.6%)	2 (4.5%)	0 (0%)	0 (0%)
**After several acute exacerbations**	13 (29.5%)	9 (20.5%)	10 (22.7%)	10 (22.7%)	2 (4.5%)
**At the end-of-life**	7 (15.9%)	2 (4.5%)	7 (15.9%)	12 (27.3%)	16 (36.4%)
**Other**	33 (75%)	2 (4.6%)	4 (9.1%)	2 (4.5%)	3 (6.8%)

COPD, Chronic obstructive pulmonary disease.

The main barriers to referral of COPD patients to specialized PC services reported by 23 participants (41.1%, *N* = 56) were the perception that patients do not want or would not like to be referred to PC and a lack of knowledge about available services (41.1%, *N* = 56). Another reason was a different perception of the PC needs of a patient between nurses and physicians.

The three most important factors hindering effective communication about EoL issues with COPD patients were the “perception that the patient is not ready” (50%, *N* = 56), “lack of skills/training (overworked team)” (42.9%, *N* = 56) and “lack of time” (35.7%, *N* = 56). Other barriers cited by four participants were the uncertainty associated with the prognosis, even if the patient has a severe disease, and thus the difficulty to initiate EoL discussions with the patient.

Thirty participants (62.5%, *N* = 48) suggested to some of their COPD patients (0%–25%) to complete advance directives or develop an advance care plan. Fourteen participants (29.2%, *N* = 48) suggested that 25%–50% of their patients to complete advance directives or develop an advance care plan.

### Perceptions of palliative care for patients with COPD

Among the three most important aspects of PC for the management of COPD patients, from the perspective of participants, “discussion around patient-accepted treatments (enteral feeding, palliative sedation, mechanical ventilation, antibiotics, intensive care hospitalizations)” was the most important topic for 31 participants (55.4%, *N* = 56) followed by “discussion of an advance care plan—values and goals of care” for 30 participants (53.6%, *N* = 56) and “identification of patients for referral to specialized palliative care” for 26 participants (46.4%, *N* = 56).

Twenty-two participants (44.7%, *N* = 48) answered neutral when asked, “How difficult is it for you to discuss EoL issues with your patients?” Seventeen (35.4%, *N* = 48) said they felt comfortable discussing EoL issues with COPD patients.

### Information and content delivered to patients with COPD

Participants were asked to rate from “Never” to “Almost always > 75%” on their perception of the provision of support in the physical, psychological, social, and existential dimensions of PC according to the World Health Organization (WHO). Twenty-two participants seemed to address psychological dimensions (47.8%, *N* = 46) “Most of the time (50%–75%)” and 15 participants addressed physical dimensions (32.6%, *N* = 46). Though social dimensions were addressed “Often (25%–50%)” by 19 participants (41.3%, *N* = 46), 23 participants addressed existential dimensions “Rarely (<25%)” (50% *N* = 46).

Regarding inclusion of relatives in discussions about values and goals of care, 23 participants answered “Sometimes” (50%, *N* = 46), and 20 participants answered “Yes” (43.5%, *N* = 46), and only 3 participants answered “No” (6.52% *N* = 46). However, they highlighted that in some cases, patients don’t want to burden relatives and don’t request them to participate in those discussions.

Approximately half of the participants (44.4% *N* = 46) include other HPs in discussions about values and goals of care. Those include the patient’s pneumologist, general practitioner, respiratory therapist, home care team, home care nurses (specialized nurses in respiratory care and others), PC team, hospital nurses, and in some cases also dietician, hypnotherapist, psychologist and psychiatrist, or medical students. The reasons to invite these HPs were mostly to have a multidisciplinary approach and bring different views into the situation. Also, according to one participant’s open-ended answer “. . .all caregivers can listen, support, help, surround by listening and have an adapted and personalized response in a palliative and humanistic approach.”

### Organization of care for COPD patients

To the question “Are there specialized PC teams that could support you in the care of COPD patients?”, 25 responded “Yes” (54.3%, *N* = 46) and mentioned two PC teams (the mobile PC team that works in the Geneva University Hospitals (CoSPa) and the PC specialists who works in the canton of Geneva, the *Groupe Genevois de médecins Pratiquant les Soins Palliatifs* (GGPSP). Twenty-one participants (45.5%, *N* = 46) don’t know if there are specialized PC teams available.

According to the majority (75%, *N* = 46), a multidisciplinary team should implement PC at home. Seven participants (15.2%, *N* = 46) thought that this was the responsibility of the specialized PC team.

Regarding preferences of coordinators for home-based PC, 19 participants (41.3%, *N* = 46) thought that it should be coordinated by a specialized PC team and 16 participants (34.8%, *N* = 46) were convinced that a multidisciplinary team should coordinate PC at home.

In the open-ended questions, participants expressed that they would like to continue to work as a multidisciplinary team and keep family and/or HPs meetings to discuss patients’ and families’ objectives. Participants also added that they would like to improve the identification of patients in need of PC in the future. Some suggested to add a tab to the electronic patient file in which it would be possible to access information on the patient’s home care. Others would like to have a PC physician assisting with medical visits in the ward. A coordinator of care was also mentioned as an added value person who would facilitate discharge and follow-up. Participants, mainly nurses, expressed that they would like to call the PC mobile team without the need for a medical prescription, which is currently mandatory.

We asked our participants about their needs for enhancing their PC skills in managing COPD patients. Their needs mostly point to (a) training in general PC, (b) knowledge about the PC resources available, (c) more time to spend with the patient, d) more time to discuss patient cases with the team, (e) easy access to PC teams, (f) coordination of care, (g) feedback about PC treatments initiated and (h) means of identification of patients in need of PC (instruments, guidelines, decision-making algorithms, etc.).

To the open-ended question “What do you think needs to be developed in the future in the palliative care management of COPD patients?”, participants answered that having a coordinator for each patient who oversees the situation, organizes multidisciplinary consultations, and coordinates the relationship between the palliative team and the pneumologist could be helpful. Communication about good care options, easily available specialized palliative care consultation, development of a safe discharge process, and availability of in-hospital care (dedicated PC units) should be available. Cited facilitators would be, (a) to have a more systematic PC consultation in some advanced cases and in the early stages of the disease, (b) public information campaign to change patients’ perceptions of PC, (c) to facilitate interactions between professionals (e-mail exchanges not being always ideal), (d) interprofessional discussions and digital platforms for possible video conferences, (e) facilitate access and information to PC, (f) more training in general PC, and (g) easily accessible education for patients and informal caregivers.

## Discussion

Although several studies investigated the needs and representation of patients with COPD concerning PC,^12,21^ far less is known about HPs’ perceptions of PC.^27,35,45^ We believe this paper adds to the knowledge that will help improve the delivery and content of PC provision for patients with COPD.

Our study evaluated the representations of PC by HPs providing care to COPD patients in a wide range of settings. In this study, most participants agreed that COPD patients required and benefited from PC in the context of a silent, progressive, disabling, non-curative disease with a high impact on QoL.

The representation and understanding of the benefits of PC for COPD patients on, (a) coping with COPD; (b) emotional symptoms; (c) respiratory symptoms; (d) illness understanding; and (f) prognostic awareness were quite congruent to what has already been demonstrated.^46–48^ Despite this knowledge, only a few HPs expressed that they do discuss or address their patients to PC in our study.

Our findings confirm several barriers to initiating PC in COPD patients.^25,26^ These include HPs perception that patients are not ready for such care and concerns about undermining patients’ hope.^25,49^ Barriers to initiate PC could be divided in three domains (a) lack of education and skills to discuss or introduce PC; (b) insufficient knowledge about local resources; (c) fear of harming patients by discussing PC. This may have an important impact on the timely referral of patients with PC needs to specialized PC services.

This study acknowledges the persistence of a significant gap in training and knowledge of HPs regarding PC, which has also been found in other studies.^25,49^ This finding is corroborated by Atreya et al. in their survey exploring the perception of respiratory physicians and their current practice of integrating PC for adult patients with chronic advanced respiratory diseases, in which around 70% of the participants (*N* = 121) felt the need for training in EoL care.^50^ This gap highlights the need for targeted educational programs and professional development opportunities to equip HPs with the necessary skills to identify and deliver effective PC and thus improve the QoL of COPD patients.

In our study, participants recognized not using or knowing the existence of tools or scales to identify PC patients and their needs such as the ESAS, SPICT™, or Pallia 10 CH.^20,51–53^ This then makes it harder to introduce PC as recommended by the European Respiratory Society clinical practice guideline, which is when physical, psychosocial, social, or existential needs are identified through a holistic needs assessment.^19^

Participants expressed uncertainty about the appropriate timing to introduce PC into the patient’s care plan. In addition, they highlighted that prognostic uncertainty and variations in disease progression make it difficult for HPs to know when to initiate PC discussions. This difficulty is further compounded by the well-recognized challenge of identifying specific transition points in patients living with COPD.^54,55^ These factors collectively emphasize the complexities in addressing and referring patients with COPD to PC.

The European Respiratory Society (ERS) clinical practice guideline, suggests that we should promote earlier PC in COPD patients.^19,56^ A recent consensus has been published on PC referral criteria for people with COPD^57^ and emphasizes that patients should be addressed to outpatient PC when they present (1) criteria for advanced respiratory therapies (2) symptoms and decision-making needs (3) a decrease in performance status, estimated by Karnofsky status and 6 min walk test, and a life expectancy of less than 6 months.^57^ In our study, 65.9% of participants never referred patients to PC, and most of them waited for advanced disease. Indeed, even the repetition of exacerbations did not prompt PC referral.^57^

The guidelines and literature support the fact that all HPs must seize every opportunity to “trigger” a palliative approach and address the patient.^54^ It has been proposed that the discussion of PC is “everybody’s job.”^22,26^ This supports the strategies to promote and involve HPs other than physicians in PC provision through educational programs and information which may then improve the diffusion of PC as stated by the recent ERS clinical practice guideline.^19^

Regarding EoL discussions, our findings show participants feel rather comfortable discussing these issues with COPD patients. These results are in contrast with other studies in which HP was not always at ease with having these discussions.^58^ Interestingly, a recent qualitative study, exploring the experience of 14 patients with advanced cancer or COPD, suggests that patients did not consider EoL discussions as being the responsibility of the pulmonologist.^59^ However, the lack of a designated clinician responsible for initiating EoL discussions with the patient can result in the absence of discussion on this topic. As it has been suggested, solutions could include embedding nurse-led PC models of care, coordinating care, having gradual and regular discussions about the evolution of the disease, and discussing long-term care plans.^60^ In fact, incorporating nurse-led PC consultations into COPD services could effectively address the unmet biological, psychological, social, and spiritual needs of COPD patients and relatives.^61,62^

There is an agreement that ACP should also be incorporated into routine COPD management.^35^ However, only a minority of participants who responded to our survey discussed or wrote ACP with patients. Barriers to ACP discussion described in the literature are: insufficient time, lack of training, and an absence of knowledge as to who is responsible for initiating ACP.^63^

As COPD progresses, patients become more homebound punctuated with exacerbations leading to hospital admissions.^64^ Consistent with other publications, most of the responders to our survey highlighted the need to integrate PC into the daily care of their patients.^65^ Pulmonary rehabilitation is a good opportunity to introduce PC as it is holistic and routine care for patients.^66^

Participants in our study were convinced that multidisciplinary care would improve the holistic management of COPD patients, especially regarding PC. Better implication of nurses in the care of patients with advanced respiratory disease was frequently mentioned, for example, as coordinators of care or initiators of PC.

### Limitations

This study has limitations as it was conducted only in the French-speaking part of Switzerland. This online survey was limited by the fact that it may be difficult to achieve adequate answers to such complex questions with only fixed-format questions and the possibility of adding open-ended comments. Employing multi-methods approaches that incorporate both quantitative and qualitative data collection techniques can provide a more comprehensive understanding of the perceptions of HPs, helping to address potential biases inherent in any single methodological approach. The voluntary nature of participation in this study poses a limitation to the generalizability of our results. Individuals who choose to participate may have already possessed a predisposition or interest in PC, introducing a recruitment bias. However, the response rate was high (57%), meaning the answers probably do represent the views of the majority of HPs working with COPD in our setting.

## Conclusion

In conclusion, this study highlights the need for more training, knowledge of existing guidelines, and information about PC. More specifically, there was an identified need to promote awareness about PC networks, tools, and scale to identify PC. Most of the barriers may come from HPs-barriers more than actual patient-barriers. The benefits of PC are well recognized. Implementation of a PC coordinator working with COPD patients may help promote timely PC referral as recommended by international guidelines.

## Supplemental Material

sj-docx-1-tar-10.1177_17534666251341748 – Supplemental material for Healthcare professionals’ representation toward optimal palliative care provision for COPD patients: a cross-sectional survey Supplemental material, sj-docx-1-tar-10.1177_17534666251341748 for Healthcare professionals’ representation toward optimal palliative care provision for COPD patients: a cross-sectional survey by Filipa Alexandra Baptista Peixoto Befecadu, Paola Gasche, Dan Adler, Ivan Guerreiro, Sophie Pautex and Lisa Hentsch in Therapeutic Advances in Respiratory Disease

sj-docx-2-tar-10.1177_17534666251341748 – Supplemental material for Healthcare professionals’ representation toward optimal palliative care provision for COPD patients: a cross-sectional survey Supplemental material, sj-docx-2-tar-10.1177_17534666251341748 for Healthcare professionals’ representation toward optimal palliative care provision for COPD patients: a cross-sectional survey by Filipa Alexandra Baptista Peixoto Befecadu, Paola Gasche, Dan Adler, Ivan Guerreiro, Sophie Pautex and Lisa Hentsch in Therapeutic Advances in Respiratory Disease

## Declarations

**Ethics approval and consent to participate:** This study obtained ethical approval from the institutional review board (*Conseil d’évaluation des études ne relevant pas de la commision d’éthique de la recherche (CEENCER)*) of the Geneva University Hospitals (2021-01). Participants were informed about the voluntary nature of their participation, the confidentiality of their responses, and their right to withdraw at any time without consequences. The email invitation notified participants that by opening the survey link and completing the survey, they acknowledged to have read the information provided and agreed to take part in the study.

**Consent for publication:** Not applicable.

**Availability of data and materials:** The data included in this study cannot be shared as participants did not consent to their data being shared.
