# Supplementary material for: Healthcare professionals’ representation toward optimal palliative care provision for COPD patients: a cross-sectional survey
Source: Ther Adv Respir Dis. 2025 May 28;19:17534666251341748. doi: 10.1177/17534666251341748 (PMC12120299; doi:10.1177/17534666251341748)
Supplement: sj-docx-1-tar-10.1177_17534666251341748 – Supplemental material for Healthcare professionals’ representation toward optimal palliative care provision for COPD patients: a cross-sectional survey [file sj-docx-1-tar-10.1177_17534666251341748.docx]

Appendix 1 - Questionnaire

Please complete the survey below. Thank you!

Age

Gender male female

Other/does not wish to answer

Profession physician physiotherapist

nurse

ASSC

Nursing assistant Other

Other profession

Do you have a specialization? yes no

if yes, which one?

In which setting do you work ? in hospital outpatient Private practice

Geneva Lung League

Other

Other

How many years have you been caring for patients with COPD < 1 year or other chronic respiratory diseases? 1– 3years

3 - 5 years

>5 years

Have you received training in palliative care in the past? Yes

No

Can you specify ?

Have you worked in a palliative care unit in the past? Yes

No

How long? (time in months)

Before answering the questionnaire, please note the distinction between general palliative care and specialized palliative care.

General Palliative Care

This refers to care provided by non-specialized healthcare professionals, based on their skills and resources.

Specialized Palliative Care

This refers to care provided by healthcare professionals specialized in palliative care, in the context of a disease and/or a situation where the patient and their loved ones are facing high complexity and instability.

The questions below refer to patients with COPD.

Section 1 - Identifying Palliative Care Needs in COPD Patients

In your opinion, can palliative care help : To relieve physical, psychological, (Select the 3 most important ones.) and spiritual symptoms

To improve quality of life and have a positive influence on the disease trajectory

To discuss the prognosis

To initiate discussions on advance care planning

To improve patients' understanding of the disease

To provide support to informal caregivers (respite/education)

To manage the end-of-life phase

Other

In your opinion, do patients with COPD require palliative care? yes

No

Maybe

Why?

Have you ever initiated a palliative care approach/ Yes.Please explain.

management for COPD patients? Sometimes. Please explain. . No. What was the reason?

Complete with your answer

Do you use guidelines/scales/tools to identify the palliative care yes

needs of COPD patients? no

Which ones?

Why?

From the criteria listed below, please indicate which ones you use (and how frequently) in your practice to identify the need for palliative care management for COPD patients:

Clinical experience

First hospital admission for an acute COPD exacerbation

Never Rarely Sometimes Often Always

Second hospital admission for an acute COPD exacerbation

Repeated hospital admissions for COPD exacerbations

Hypoxemia requiring home oxygen therapy

Hypercapnia

Need for non-invasive ventilation (NIV)

during hospitalization

Need for home care for basic needs

Elderly patient

Patient with severe comorbidities

Low FEV1 (< 30%)

Low BMI, unexpected weight loss, or cachexia

High BODE score

High score on the CAT (COPD Assessment Test™)

High score on the MRC Dyspnea

Questionnaire

Patient's desire

None of the mentioned criteria

Other

Please name any other criterion not described.

How often do you encounter the following symptoms in patients with COPD??

Dyspnea

Pain

Anxiety Depression Fatigue Nausea Drowsiness

Other symptom

Never Rarely

(<25%)

Often

(25-50%)

Most of the time (50-75%)

Almost Always

(>75%)

Please name any other symptom.

At what point do you refer COPD patients to specialized palliative care (outpatient/inpatient/home care)??

At the time of diagnosis

After the first acute exacerbation

Never Rarely

(<25%)

Often

(25-50%)

Most of the time (50-75%)

Almost Always

(>75%)

After multiple acute exacerbations

At the end of life

Other time

Please specify at what other time.

Which of the factors described below have been a In my region/hospital, there is no palliative care barrier for you in referring COPD patients to palliative care? Team vailable.

It is difficult to obtain appointments.

My patients do not like or do not want to be

Referred to palliative care

I feel like I am abandoning my patients by referring them to palliative care

I cannot find the contact information for the specialized palliative care consultation.

Lack of knowledge about available resources

Other

If other, please specify below.

What are the factors that constitute barriers to communicating Lack of time

with COPD patients about end-of-life care? Lack of skills/training (overburdened team)

(Select the 3 most important ones)

Lack of support from governing organizations

Fear of harming the patient’s hope Perception that the patient is not ready No obstacles

Other

If other, please specify below.

What proportion of COPD patients under your care have you 0-25%

Suggested drafting advance directives or advance care plan for 25-50%

50-75% 75% >75%

Section 2 - Perceptions of Palliative Care for COPD Patients

In your opinion, what are the most important aspects Identifying patients for referral to palliative of palliative care for COPD patients? care (specialized?)

( Select the 3 most important ones) Discussion about prognosis

Advance care planning discussions – values and care goals

Advance directives – drafting/updating

Discussion about resuscitation

Discussion about treatments accepted by the patient (enteral feeding, palliative sedation, mechanical ventilation, antibiotics, ICU hospitalizations)

Organization of palliative home care

Defining the care coordinator (pulmonologist, primary care physician, specialized nurse, etc.)

Information transfer between pulmonologist – general practitioner – care coordinator

Admission for hospitalization during nights/weekends

Other

If other, please specify below.

What are the major challenges you encounter in your practice when dealing with a COPD patient with palliative care needs?

How difficult is it for you to discuss end-of-life very difficult issues with your patients? Difficult

Neutral

I feel comfortable

Section 3- Information and content delivered to COPD patients

According to the WHO, palliative care focuses on the physical, psychological, social and existential dimensions. To what extent do you provide support in each of these dimensions?

Physical (e.g. pain management)

Never Rarely

(<25%)

Often

(25-50%)

Most of the time (50-75%)

Almost always

(>75%)

Psychological (e.g., anxiety management)

Social (e.g. support for caregivers)

Existential (e.g., spiritual support)

When you initiate discussions about values and care goals, yes

do you include the patient relatives ? no

sometimes

Why?

When you initiate discussions about values and care goals, yes do you include other healthcare professionals ? No

sometimes

Which healthcare professionals?

Why?

In your opinion, what should be developed in the future for the palliative care management of COPD patients?

Are there specialized palliative care teams that could support palliatifs yes you in the care of COPD patients? No

I don’t know

If yes, which team ?

In your opinion, who should implement palliative care at home? Pulmonologist

(Select one answer only) general practitioner

Specialized palliative care team

The physician in charge of the patient at discharge (if hospitalized)

Multidisciplinary team (pulmonologist, primary care physician, coordinating nurse)

Other

If other, please specify below.

In your opinion, who should coordinate palliative care at home ? Care provider

(Select one answer only) Pulmonologist

General practicioner

Specialized palliative care team

The physician in charge of the patient at discharge (if hospitalized)

Multidisciplinary team (pulmonologist, primary care physician, coordinating nurse)

Coordinating nurse

Other

If other, please specify below.

How would you like to collaborate with specialized palliative care teams in the future?

What do you need to enhance your palliative care skills for patients with COPD?

Would you be interested in participating in a short yes

Training session (approximately 1.5 hours) on No

identifying patients in need of palliative care or

drafting advance directives?

If yes, in what format?

If you have any comments or suggestions, please write them below.
