# Supplementary material for: Healthcare professionals’ representation toward optimal palliative care provision for COPD patients: a cross-sectional survey
Source: Ther Adv Respir Dis. 2025 May 28;19:17534666251341748. doi: 10.1177/17534666251341748 (PMC12120299; doi:10.1177/17534666251341748)
Supplement: sj-docx-2-tar-10.1177_17534666251341748 – Supplemental material for Healthcare professionals’ representation toward optimal palliative care provision for COPD patients: a cross-sectional survey [file sj-docx-2-tar-10.1177_17534666251341748.docx]

Criteria Used in Clinical Practice to Identify the Need for Palliative Care in COPD Patients

| From the criteria listed below, please note which ones you use (and how often) in your practice to identify the need for palliative care management of COPD patients: | | | | | |
| --- | --- | --- | --- | --- | --- |
|  | Never | Rarely | Sometimes | Frequently | Always |
|  | N Percent | N Percent | N Percent | N Percent | N Percent |
| Clinical experience  N= 46 | 3 (6.5%) | 3 (6.5%) | 2 (4.4%) | 17 (37%) | 21 (45.7%) |
| First admission to hospital for acute COPD exacerbation of  N= 45 | 22 (48.9%) | 14 (31.1%) | 5 (11.1%) | 4 (8.9%) | 0 (0.0%) |
| Second hospital admission for acute COPD exacerbation  N= 44 | 13 (29.6%) | 13 (29.6%) | 12 (27.3%) | 4 (9.1%) | 2 (4.5%) |
| Repeated hospital admissions for COPD exacerbation  N= 46 | 8 (17.4%) | 1 (2.2%) | 9 (19.6%) | 17 (37%) | 11 (23.9%) |
| Hypoxemia with need for oxygen therapy at home N= 46 | 10 (21.7%) | 4 (8.7%) | 12 (26.1%) | 15 (32.6%) | 5 (10.9%) |
| Hypercapnia  N= 45 | 13 (28.9%) | 5 (11.1%) | 12 (26.7%) | 10 (22.2%) | 5 (11.1%) |
| Need for NIV during hospitalization  N= 45 | 13 (28.9%) | 6 (13.3%) | 16 (35.6%) | 7 (15.6%) | 3 (6.6%) |
| Need for home care for basic care N= 45 | 11 (24.4%) | 6 (13.3%) | 8 (17.8%) | 18 (40%) | 2 (4.4%) |
| Elderly patient  N= 45 | 9 (20%) | 8 (17.8%) | 12 (26.7%) | 13 (28.9%) | 3 (6.7%) |
| Patient with severe comorbidities  N= 45 | 7 (15.6%) | 4 (8.9%) | 7 (15.6%) | 18 (40%) | 9 (20%) |
| Low FEV1 (<30%)  N= 45 | 10 (22.2%) | 6 (13.3%) | 11 (24.4%) | 16 (35.6%) | 2 (4.4%) |
| Low BMI or unexpected weight loss or cachexia  N= 45 | 8 (17.8%) | 6 (13.3%) | 13 (28.9%) | 16 (35.6%) | 2 (4.4%) |
| High BODE score  N= 45 | 16 (35.6%) | 3 (6.7%) | 10 (22.2%) | 14 (31.1%) | 2 (4.4%) |
| High CAT score  N= 45 | 16 (35.6%) | 4 (8.9%) | 15 (33.3%) | 8 (17.8%) | 2 (4.4%) |
| High score in the mMRC questionnaire  N= 45 | 14 (31.1%) | 4 (8.9%) | 12 (26.7%) | 13 (28.9%) | 2 (4.4%) |
| Desired by the patient  N= 45 | 6 (13.3%) | 5 (11.1%) | 5 (11.1%) | 5 (11.1%) | 24 (53.3%) |
| None of the criteria mentioned  N= 44 | 40 (90.9%) | 1 (2.3%) | 2 (4.6%) | 1 (2.3%) | 0 (0.0%) |
| Other  N= 35 | 32 (91.4%) | 0 (0.0%) | 2 (5.7%) | 0 (0.0%) | 1 (2.9%) |

NIV: non-invasive ventilation, FEV1: Forced Expiratory Volume in the first second, BMI : Body Mass Index, BODE: Body mass index, airflow Obstruction, Dyspnea, and Exercise capacity, CAT: COPD Assessment Test, mMRC : modifed Medical Research Council
